# Supplementary material for: Interpretable video-based tracking and quantification of parkinsonism clinical motor states
Source: NPJ Parkinsons Dis. 2024 Jun 25;10:122. doi: 10.1038/s41531-024-00742-x (PMC11199701; doi:10.1038/s41531-024-00742-x)
Supplement: Supplementary file 1 — Supplementary Information [file 41531_2024_742_MOESM1_ESM.pdf]

Supplementary Information

Supplementary Table 1: Effect Sizes of Selected Features in Best Performing Model (LR).

Feature	Coefficient (β)	Odds Ratio (OR)
Pinky Mvmt. Speed [Rel. Pwr. >6 Hz]	1.39 ± 0.78	4.02 ± 2.18
Ankle Separation [Rel. Pwr in 1-2 Hz]	0.80 ± 0.33	2.22 ± 1.39
Ankle Separation [Rel. Pwr. in 0.5-1 Hz]	0.73 ± 0.33	2.07 ± 1.39
Finger Tapping Speed [Rel. Pwr in 4-6 Hz]	0.61 ± 0.37	1.83 ± 1.45
Arm-Body Ang. Vel. [Rel. Pwr in 0.5-1 Hz]	0.57 ± 0.35	1.77 ± 1.42
Pinky Mvmt. Speed [Half Pwr. Freq.]	-0.34 ± 0.74	0.71 ± 2.10
Δ Ankle-Hip Dist. [Rel. Pwr. in 2-4 Hz]	-0.64 ± 0.33	0.53 ± 1.39
Finger Tapping Amp. [MAD]	-0.73 ± 0.33	0.48 ± 1.39
Δ Ankle Separation [Rel. Pwr. in 4-6 Hz]	-0.93 ± 0.33	0.40 ± 1.39
Arm-Body Ang. Vel. [Rel. Pwr in 4-6 Hz]	-1.16 ± 0.35	0.31 ± 1.42
Finger Tapping Speed [Rel. Pwr. in 2-4 Hz]	-1.23 ± 0.39	0.29 ± 1.48
Ankle-Hip Dist. [STD]	-1.55 ± 0.43	0.21 ± 1.54
Neck Angle [Rel. Pwr. in 2-4 Hz]	-2.07 ± 0.59	0.13 ± 1.80

LR = Logistic Regression. OR = e^{β} represent the effect sizes of features. For each unit of increase in the corresponding standardized feature, OR is the multiplicative factor to the odds of the subject having higher motor deficits. β and OR are presented as the mean estimate ± 95% confidence interval.

Supplementary Table 2: Temporal and spectral metrics used to characterize movement measurements extracted from video recordings.

	Metric	Formulation
Temporal	Mean	$\mu = \frac{1}{T}\sum_{t=t_0}^{t_{T-1}} x[t]$
	Standard deviation (STD)	$\sigma = \sqrt{\frac{1}{T}\sum_{t=t_0}^{t_{T-1}}(x[t]-\mu)^2}$
	Median absolute deviation (MAD)	$MAD = Median(\lvert x[t]-\mu\rvert)$
	Skewness	$\frac{m_3}{{m_2}^{3/2}}$ where $m_i = \frac{1}{T}\sum_{t=t_0}^{t_{T-1}}(x[t]-\mu)^i$
	Kurtosis (Tailed-ness; amount of outliers/extreme values)	$\frac{1}{T\sigma^4}\sum_{t=t_0}^{T t_{T-1}}(x[t]-\mu)^4$
Spectral	Half-Power Frequency	ω^* s.t. $\sum_{\omega=\omega_0}^{\omega^*} S_x(\omega) = \sum_{\omega=\omega^*}^{\Omega} S_x(\omega)$
	Spectral Entropy	$-\sum_{\omega=\omega_0}^{\Omega} s(\omega)\ ln\ s(\omega)$ where $s(\omega) = \frac{S_x(\omega)}{\sum_{\omega=\omega_0}^{\Omega} S_x(\omega)}$
	Relative Powers (in 0.5-1, 1-2, 2-4, 4-6, >6 Hz frequency bands)	$\frac{\sum_{\omega=\omega_a}^{\omega_b} S_x(\omega)}{\sum_{\omega=\omega_0}^{\Omega} S_x(\omega)}$

$x[t]$ denotes the discrete movement time series at time t.
$S_x(\omega)$ denotes the power spectral density of x.

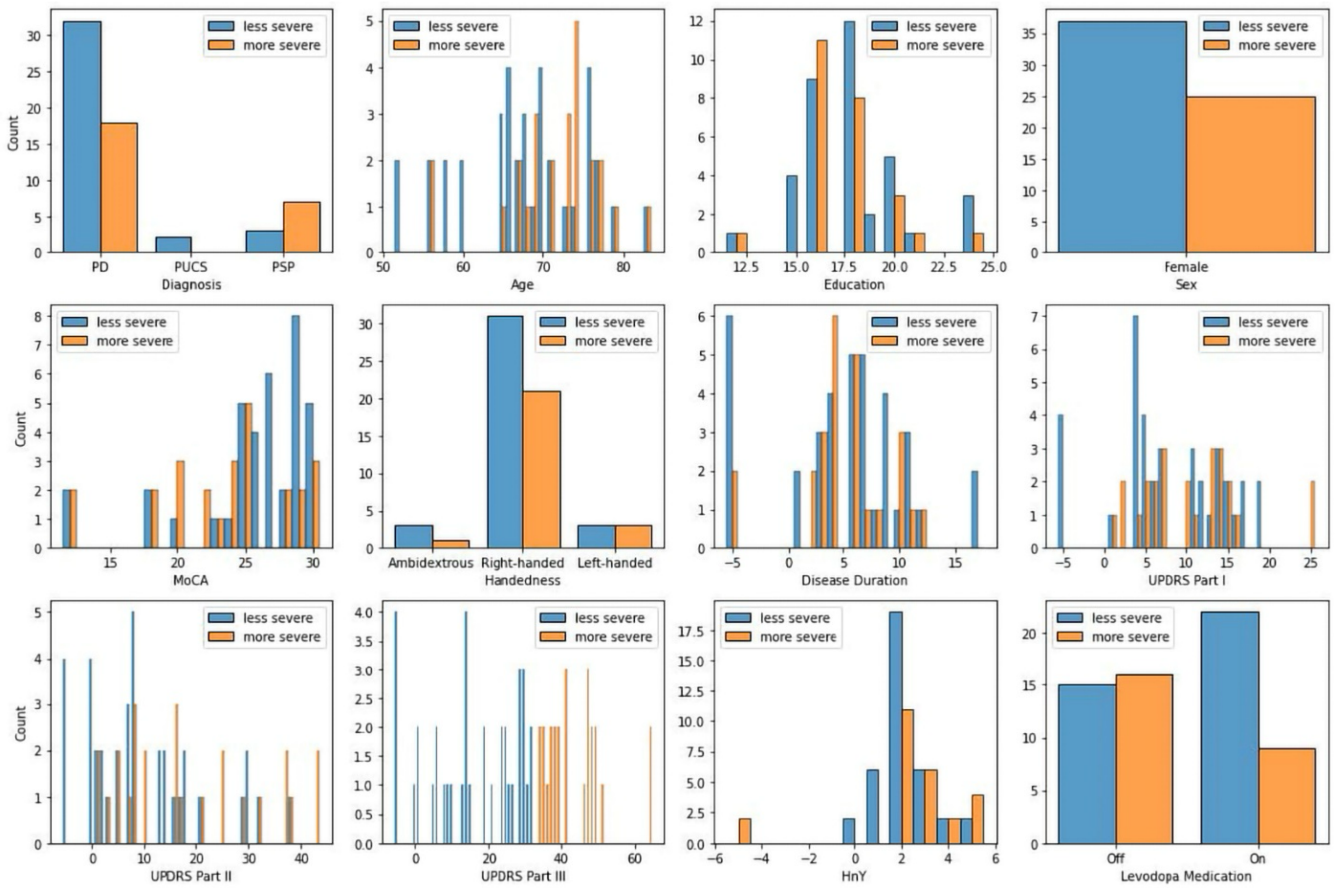

Supplementary Figure 1: Histograms showing distributions of clinical and demographic characteristics between low and high motor symptom severity groups.

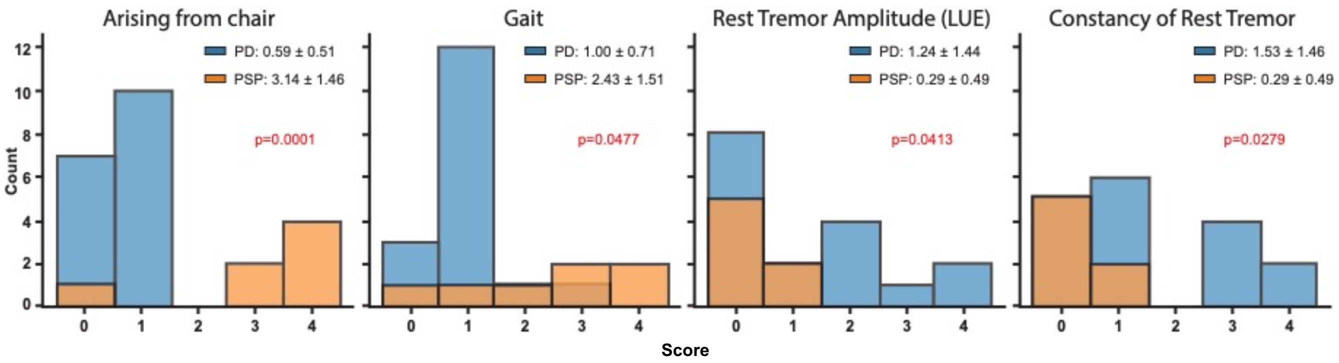

Supplementary Figure 2: UPDRS Part III subscores with significant differences between PD and PSP patients of the same severity group. LUE = left upper extremities. Both PD and PSP patients belong to the dichotomized group with higher motor impairment. Significant differences ($p < 0.05$) in sample means is determined via a Permutation Test (100,000 repetitions).

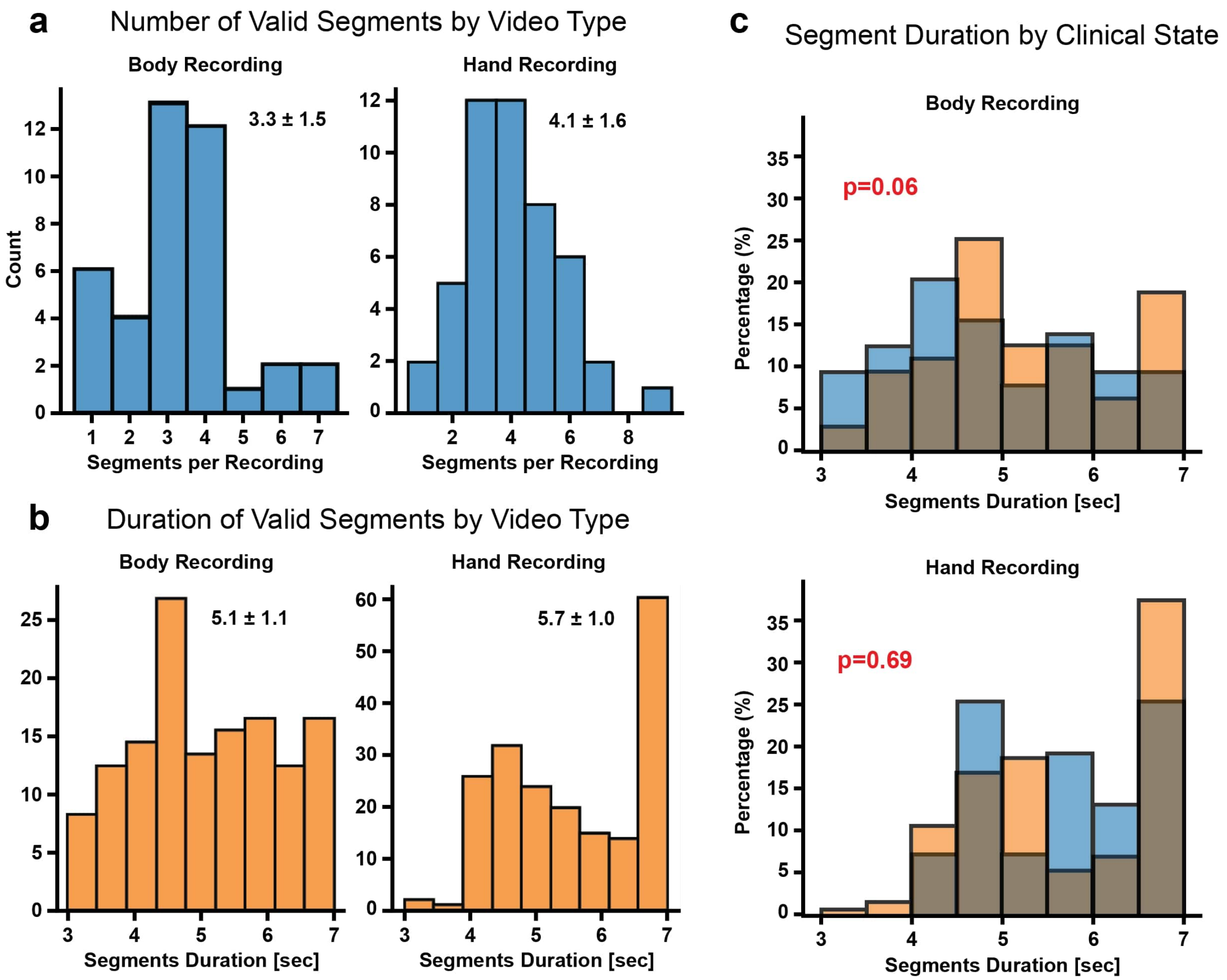

Supplementary Figure 3: Histograms showing distributions of video segment statistics. a-b. Time segment counts per recording and their durations are roughly normally distributed. **c.** There are no statistically significant ($p < 0.05$) differences in video segment duration by clinical states in either segments derived from body recordings (top) or segments derived from hand recordings (bottom). Sample means are compared using a two-sample *t*-test.

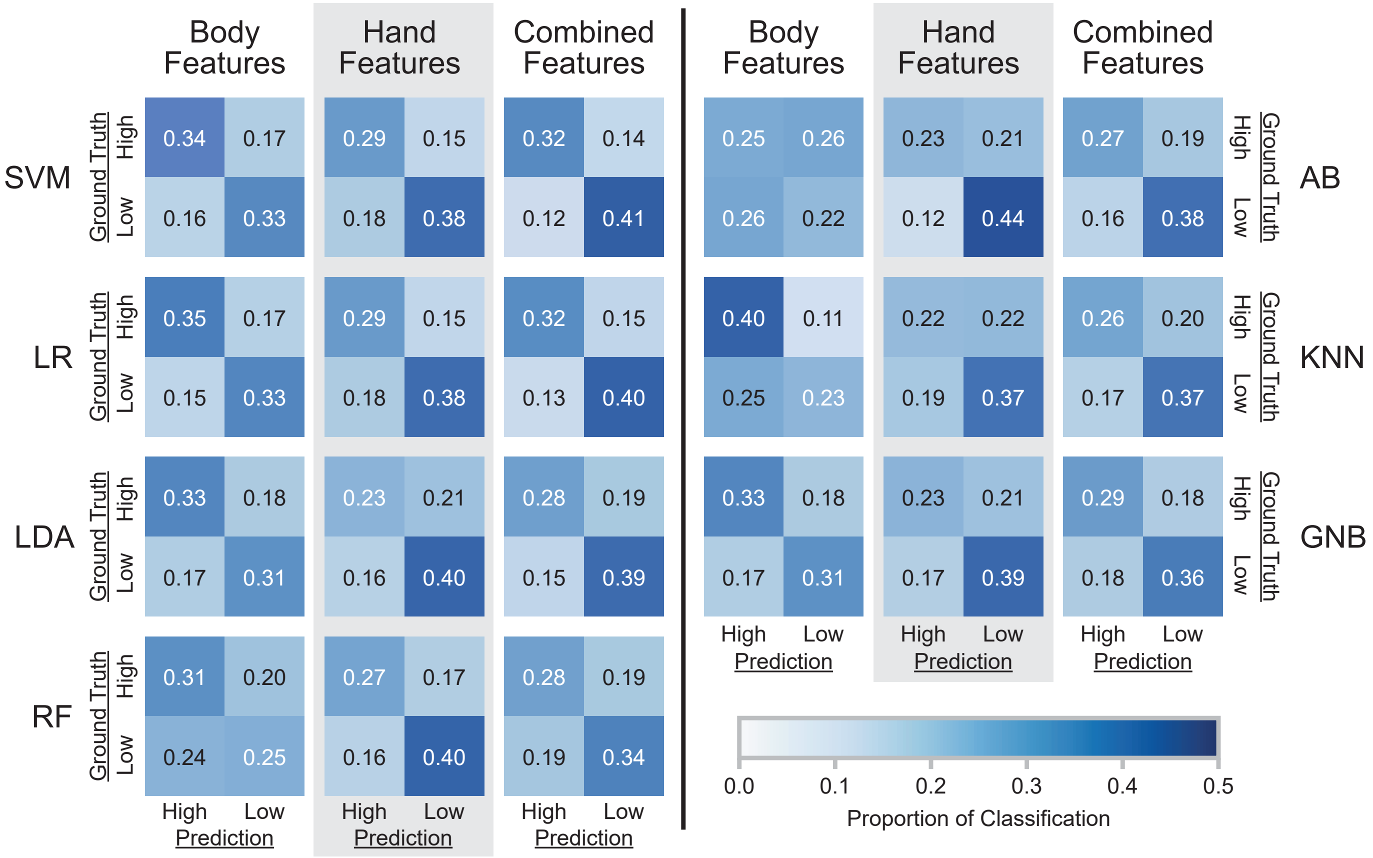

Supplementary Figure 4: Average confusion matrices for selected classifiers. Numbers in the confusion matrices represent proportions of classification results that are: (top left) true positive; (top right) false negative; (bottom left) false positive; (bottom right) true negative. Compared to classifiers trained with gait features, classifiers trained with finger-tapping features misclassified the high motor impairment class more. Classifiers trained with both gait and finger-tapping features had more confidence and accuracy when predicting the motor impairment labels. LDA = linear discriminant analysis; LR = logistic regression; SVM = support vector machine; RF = random forest; AB = adaptive-boosted trees; KNN = K-nearest neighbors; GNB = Gaussian naive Bayes.

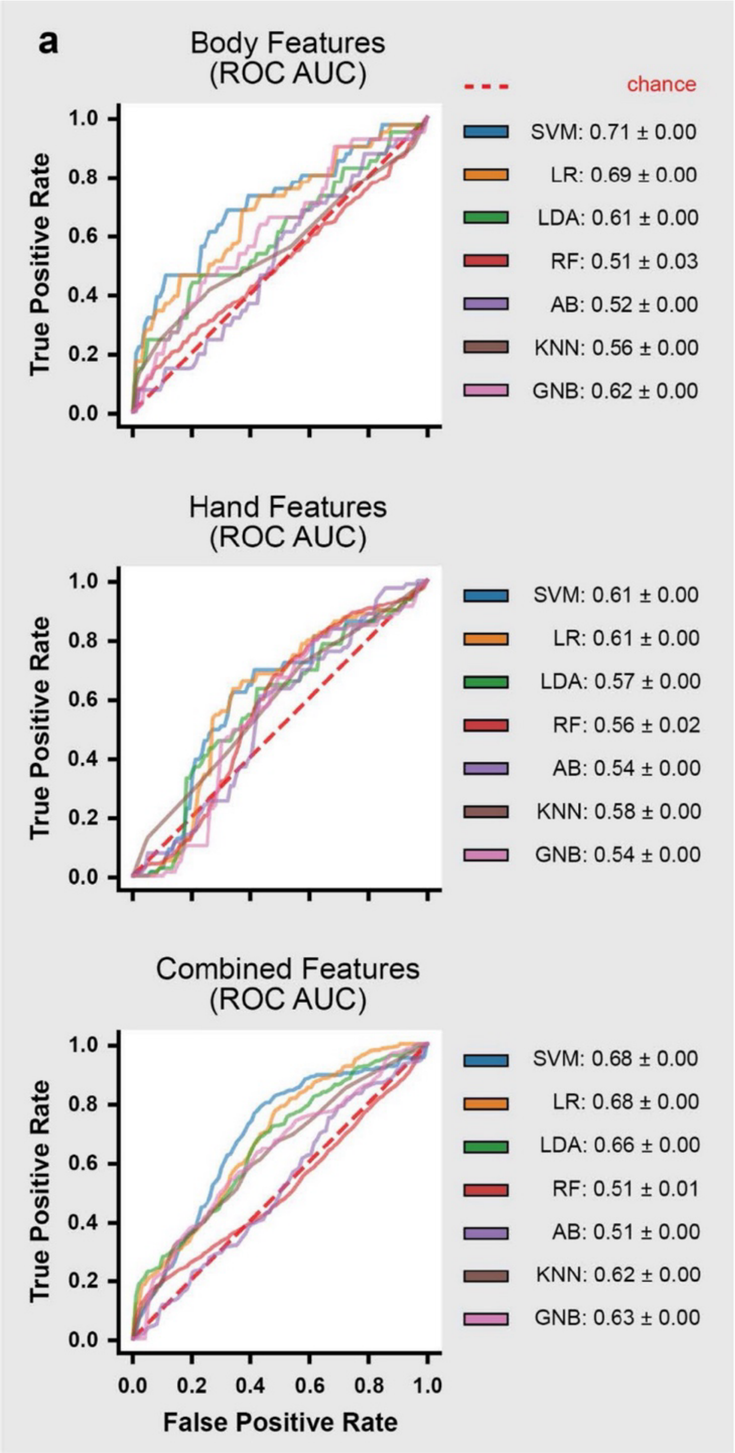

Supplementary Figure 5: Classification performances of PD samples only. SVM = support vector machine; LR = logistic regression; LDA = linear discriminant analysis; RF = random forest; AB = adaptive-boosted trees; KNN = K-nearest neighbors; GNB = Gaussian naive Bayes; ROC AUC = area under receiver operating characteristics curve. For the body feature dataset, we dichotomized the samples into 65 low- and 41 high-severity groups. For the hand feature dataset, we dichotomized the samples into 83 low- and 79 high-severity groups. For the combined dataset, there are 417 low- and 334 high-severity samples.

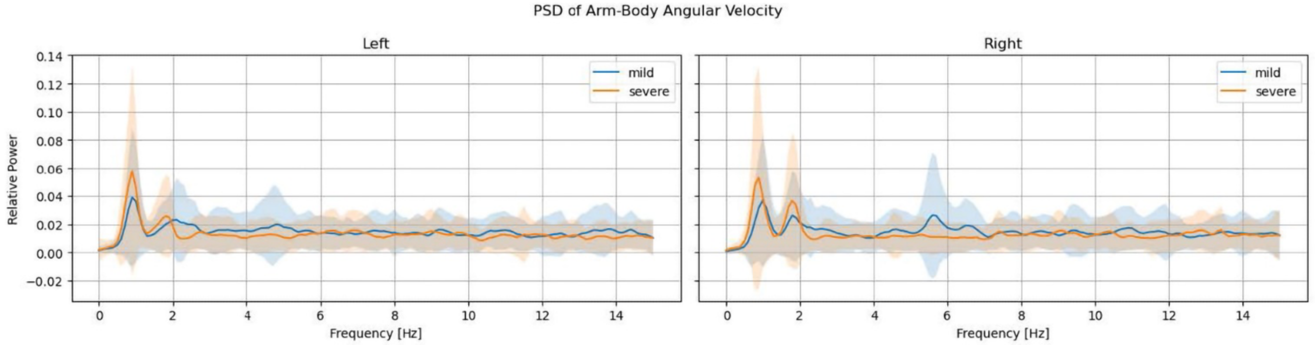

Supplementary Figure 6: Average relative PSDs of arm-body lateral angular velocity of all participants. The shaded areas correspond to ± 1 standard deviation.

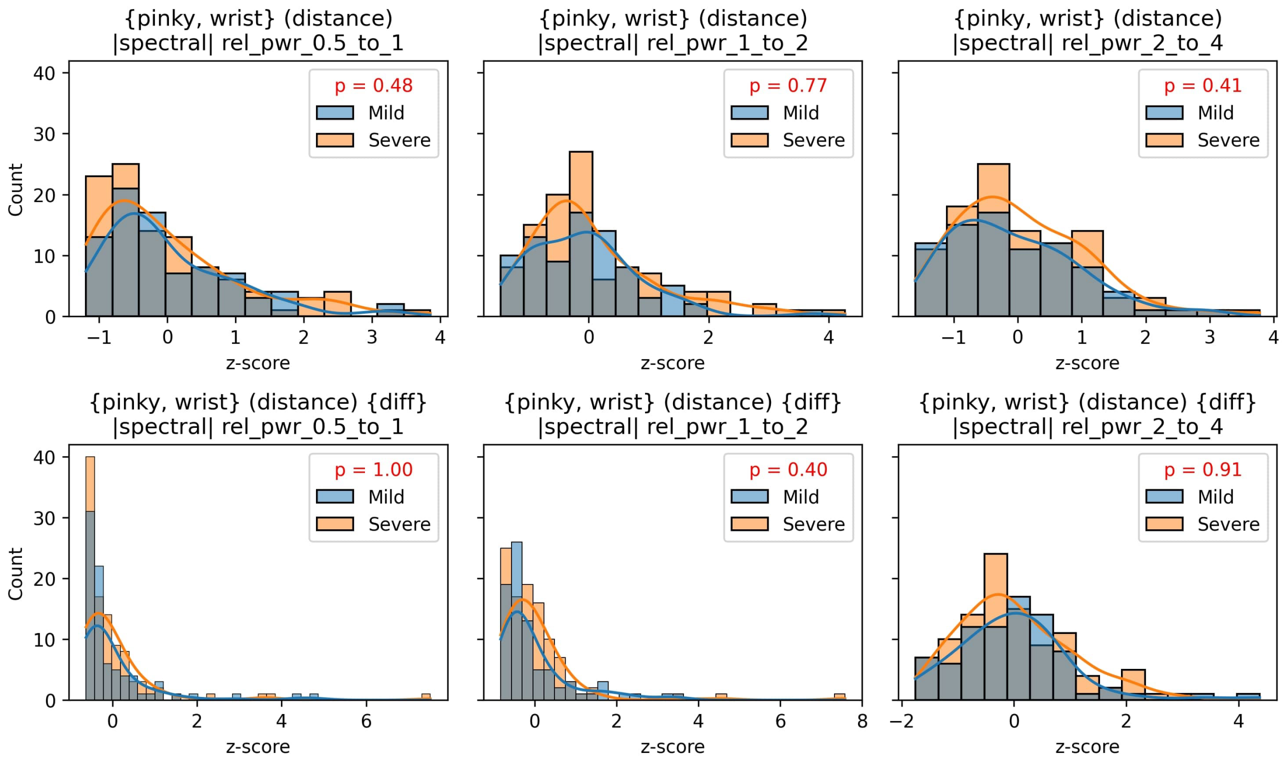

Supplementary Figure 7: Pinky movement features of at-rest hand show no significant difference between low and high motor symptom severity groups in low frequency ($< 4\ Hz$) regimes. Two-sided Mann–Whitney U test; significance threshold $p = 0.05$. Movement features for other digits of the resting hand also showed no statistically significant differences between the severity groups in these regimes. These results suggest that the main contributor to differential high frequency pinky movement patterns between PD severity states is likely not dyskinesia but intrinsic sharp movements due to loss of movement fluidity.

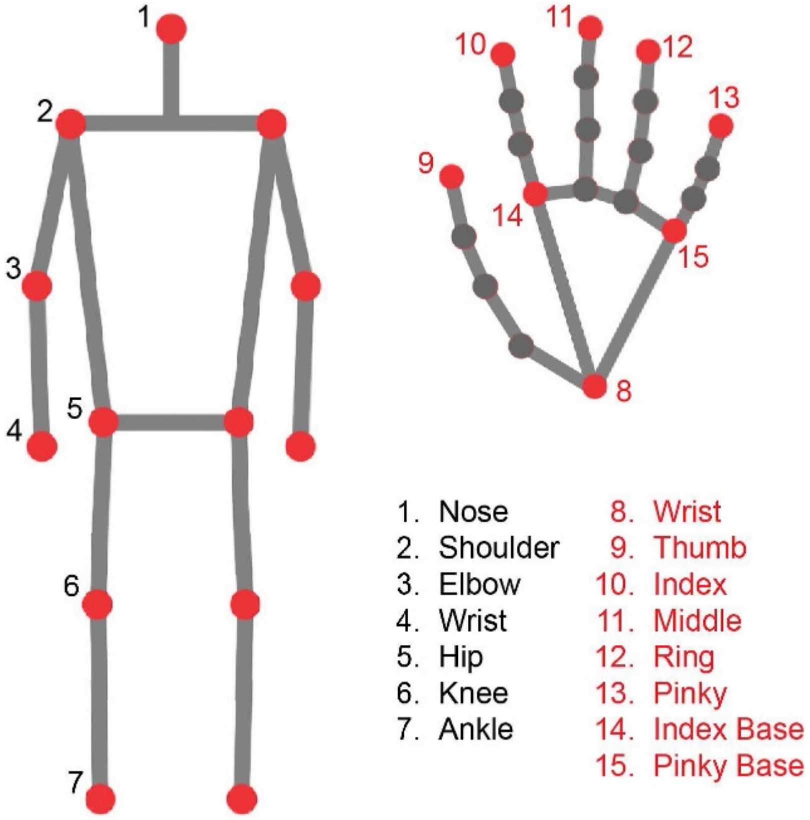

Supplementary Figure 8: Body and hand skeletons with key landmarks used in analysis labeled. For simplicity, we only labeled relevant landmarks on one side of the body and one of the hands. Some labels were renamed and differed from the official nomenclatures provided by MediaPipe.

Supplementary Video 1: Visualizations of extracted landmark kinematics of an example pose recording. At any point in the videos, a green background indicates that the given frame is error-free and usable, whereas a red background indicates that the frame is to be discarded.

Supplementary Video 2: Visualizations of extracted landmark kinematics of an example hand recording. At any point in the videos, a green background indicates that the given frame is error-free and usable, whereas a red background indicates that the frame is to be discarded.
